# Ecological processes of bacterial microbiome assembly in healthy and dysbiotic strawberry farms

**DOI:** 10.1186/s12870-024-05415-8

**Published:** 2024-07-20

**Authors:** Dominika Siegieda, Jacek Panek, Magdalena Frąc

**Affiliations:** Institute of Agrophysics, Polish Academy of Sciences, Doświadczalna 4, Lublin, Lublin, 20-290 Poland

**Keywords:** Bacterial community, Dysbiosis, Ecolocical health biomarkers, Eubiosis, Plant holobiont, Soil metataxonomy, Soterobiont

## Abstract

The bacterial microbiome plays crucial role in plants’ resistance to diseases, nutrient uptake and productivity. We examined the microbiome characteristics of healthy and unhealthy strawberry farms, focusing on soil (bulk soil, rhizosphere soil) and plant (roots and shoots). The relative abundance of most abundant taxa were correlated with the chemical soil properties and shoot niche revealed the least amount of significant correlations between the two. While alpha and beta diversities did not show differences between health groups, we identified a number of core taxa (16–59) and marker bacterial taxa for each healthy (Unclassified Tepidisphaerales, Ohtaekwangia, Hydrocarboniphaga) and dysbiotic (Udaeobacter, Solibacter, Unclassified Chitinophagales, Unclassified Nitrosomonadaceae, Nitrospira, Nocardioides, Tardiphaga, Skermanella, Pseudomonas, Allorhizobium-Neorhizobium-Pararhizobium-Rhizobium, Curtobacterium) niche. We also revealed selective pressure of strawberry rhizosphere soil and roots plants in unhealthy plantations increased stochastic ecological processes of bacterial microbiome assembly in shoots. Our findings contribute to understanding sustainable agriculture and plant-microbiome interactions.

**Supplementary information:**

The online version contains supplementary material available at 10.1186/s12870-024-05415-8.

## Introduction

Strawberry (*Fragaria* × *ananassa* Duch.) is a crucial berry crop in global agriculture, with China and Poland being important exporters of this fruit and possessing the largest cultivation areas worldwide as of 2020 [1]. However, the delicate cell wall structure of strawberry fruit renders it susceptible to pathogen intrusion, resulting in compromised fruit quality and decreased production [2]. As the susceptibility of strawberry to pathogenic microorganisms decreases the productivity of these crops, organic farming’s potential for the reduction of chemical pesticides and restoring natural biodiversity of the micro and mycobiomes in the agriculture, could promote plant health and productivity, while being respectful to the environmental biodiversity.

Organic farming [3], including organic strawberry production, is increasing its market share year by year [1], as being more sustainable and environmentally friendly. This is caused by the reduction of pesticide and chemical fertilizers use, simultaneously improving the productivity and plant health which in turn creates more opportunities to explore and understand the role of microbiomes in promoting plant growth and yield. This reduction has a potential to restore the natural biodiversity of microorganisms, that indeed promote plant health and enhance agricultural yields [4], as the microbiome plays an important role in disease suppression, nutrient cycling, enhancing stress tolerance and ecosystem functioning in the agriculture, with the importance of the plant holobiome concept [5–8]. Emphasizing the relevance of the plant holobiome concept, this underscores the intricate web of interactions within the plant-microorganism relationship. Some of the key factors influencing soil and plant health are bacteria, fungi and archea that are crucial components of host-microorganism interactions [9]. Among these, plant-associated bacteria, such as endophytic bacteria inhabiting plant tissues, rhizobacteria in the rhizosphere soil, and epiphytic bacteria on the phyllosphere, have been extensively studied. Some of the well-known examples of this beneficial symbiotic relationships between bacteria and plants are *Rhizobium* spp. with legume crops, where bacteria fix atmospheric nitrogen, increasing its availability in the bulk soil [10]. Another example are phosphorous solubilizing microbes (PSM), that produce various organic acids that help mineralize fixed phosphorous into plant-available forms [11]. Bacteria also can provide indirect benefits to the host plant by competing with pathogens for resources or producing various antibiotics, lytic enzymes and volatile compounds that act against them [12]. Also, bacteria indirectly influence the plant health by maintaining soil structure, performing organic matter decomposition and reducing soil erosion and improving water-holding capacity [13, 14]. Additionally, plant-microbe interactions induce defence responses such as induced systemic resistance (ISR) and systemic acquired resistance (SAR), which help the host plants to resist phytopathogens [15].The plants take advantage of these microbial mechanisms and are able to modulate microorganism assembly for their benefit, for example with phytohormones: salicylic acid and jasmonic acid [16–19]. It had been reported, that the environmental stress can be resisted, or cause significant changes in α- and β-diversity of the plant microbiomes [20]. Anna Karenina Principle (AKP) had been proposed as to highlight the prevalence of stochastic processes of assembly in unhealthy microbiome and deterministic processes in healthy microbiomes [21]. The discovery of these intricate interactions between the host plant and microbiome surrounding it, caused a shift in the perception of the two - the holobiont concept had been proposed, where the host organism and the microbiomes are seen as one meta-organism [22, 23]. Beside the beneficial interactions, bacteria, such as *Xanthomonas fragariae* [24], can also act as phytopathogens to the strawberry plants, causing lowered yields, reducing quality of the fruit and the state of dysbiosis in the holobiont [21, 23]. Olimi et al. [25] found shifts in the reduction of microbial diversity fruit microbiome causing disease occurrence and indicated transfer of significant fractions of microbes within the phyllosphere compartments with huge biodiversity which ensured the plant health which can be considered as one of mechanisms of bacterial contributing to strawberry health. Although there have been advances in our understanding of the relationship between bacteria and their host plants, there remain significant gaps in our knowledge of the interactions between the two and the resulting impacts on the well-being of crops grown for agriculture. Many recent studies focused on greenhouse and field experiments in the context of mutual influence of bacteria and strawberry on each other, however wide range of niches hasn’t been addressed. Recent studies focused mainly on the rhizosphere soil microbiome composition [26, 27], bypassing the influence of physicochemical properties of the soil on the plant health status and microbiome composition. Moreover, although microbiome research were helpful in the discovery of harmful and beneficial microorganisms over the last years [23] and it is well known that plant-microbes interactions are important in plant health improvement, the holistic disease-preventing bacteria phenomenon still belongs to new approaches. What is more, in recent years Cervava and Berg [28] implemented the soterobiont new term to define disease-preventing microorganisms within the microbiota of higher organisms. The soterobiont includes prevention of at least one disease, is associated with at least one host organism, and has possibility to transfer disease resistance to individuals of the same species. The discovery of microorganisms that prevent disease development can provide new approaches to control plant diseases, and can be helpful in explaining certain disease resistance. Therefore, future needs include research of healthy and diseased plants in order to define microbial composition and potential processes, in holistic way inside microbiome, important in disease prevention and development of control agents. In order to deeply understand bacterial community interactions the aim of the study was to investigate the bacterial microbiome assembly in healthy and unhealthy organic farms of strawberry, located on different soil types, with the focus on soil (bulk soil and rhizosphere soil) and plant (roots and shoots) niches. We also evaluated the ecological processes that were the most important in shaping the bacterial microbiome assembly in each plant niche, to widen the understanding the relationship between health status of the strawberry plantation and bulk soil and plant niche in microbial composition assembly.

## Experimental procedures

### Sample acquisition, weather conditions and physicochemical properties of the bulk soil

In July 2019 we collected samples from 13 healthy and unhealthy organic strawberry cultivations (Supplementary Table 1) located in south-east Poland (50°N, 23°E), as described recently [29]. Unhealthy farms consisted of strawberry plants with visible leaf discoloration and necrosis (in the dysbiotic state), and healthy - without visible symptoms of the disease (Supplementary Fig. 1). Moreover, in unhealthy strawberries sympthomps of phytopathogenes (*Pestalotiopsis* sp, *Colletotrichum* sp., *Phytophthora* sp. and *Verticillium* sp.) were appeared or plants were characterized by poor growth, depending on tested farms (Supplementary Table 1). Before set up of strawberry farm, the following fertilization and biopreparations were applied: manure (40 t ha^− 1^), green fertilizers for plowing, Condit (2 t ha^− 1^), Physio Natur PKS (400–600 kg ha^− 1^), Ema Farma Plus (40–60 l ha^− 1^) and Fungilitic (10 l ha^− 1^). During growing season the following biopreparations were used: mycorrhization of the root system, and several times in growing season: Fungilitic (5–10 l ha^− 1^), Trichofit (0.3 kg ha^− 1^), Fitoprotect (1–2 kg ha^− 1^), Biofosforin (1 kg ha^− 1^), Biomag Plon (1.5 kg ha^− 1^), foliar sprays several times during season every 7–10 days X-Forte, Prev-Am 0.3–0.4% against pests. Moreover, in order to supplement the deficiencies of micro- and macroelements the following fertilizers for organic farming were applied: potassium sulphate and potassium salt (not exceeding 60 kg/ha), foliar fertilizer Olibio (2–4 l ha^− 1^), fertilizer chalk, Condit (0.5-1 t ha^− 1^), aminokwas plus (1–2 l ha^− 1^) and Physio Natur PKS (400–600 kg ha^− 1^). In fertigation system liquid biopreparations were applied several times during growing season. The information regarding the weather conditions from the nearest weather station, which included monthly maximum and minimum temperatures in 2019, daily maximum and minimum temperatures in July 2019, monthly sums of precipitation in 2019, and daily precipitation in July 2019 were also gathered (Supplementary Fig. 2A, 2B, 2 C, and 2D, respectively).

### Chemical properties of the bulk soil

The chemical properties of the bulk soil, including pH, P_2_O_5_, Mg, K_2_O, and organic carbon, were measured in Regional Chemical and Agricultural Station in Lublin (Poland) according to standard procedures and the results are presented in Supplementary Table 1. These parameters were selected because for organic strawberry production fertilization is based on the soil analyses towards the content of these elements and nutrients needs of plants and is compared with specific limit numbers of these nutrients for strawberry crops [30]. Moreover, this table includes all metadata connected with tested strawberry farms with bulk soil type, strawberry vatiety, cultivation type as well as status of plants. We utilized the chemical properties data to determine the Spearman correlations between the health status of each plant and bulk soil niche and the relative abundance of bacterial Phyla, employing the ‘phylosmith’ R package. Additionally, we examined the relationship between the chemical properties of the bulk soil and the alpha diversity of the niches using a linear regression model. Significant differences in chemical properties of the bulk soil between healthy and unhealthy farms were determined using Wilcoxon test.

### DNA isolation and Amplicon sequencing

The methods used for obtaining microbial DNA from bulk and rhizosphere soil, as well as plant niches of strawberries regarded as holobiont and the subsequent amplicon sequencing procedure have been described before [29], but here we amplified the 16 S v3v4 fragment of bacterial DNA using primers from Klindworth et al. [31].

### Amplicon data analysis

To determine the composition of the microbial community, we utilized the QIIME2 (Quantitative Insights Into Microbial Ecology) environment (version 2020.11) [32] within the Linux shell (Ubuntu ver. 20.04.3 LTS). The primer trimming was performed using Cutadapt [33], and Amplicon Sequence Variants (ASVs) were identified with DADA2 (Divisive Amplicon Denoising Algorithm v. 2) [34] and classified taxonomically with the Scikit-learn (sklearn) classifier [35] and SILVA 138 database [36]. We created a phyloseq object in the R language (v.4.0.3) using RStudio (v.1.4.2) (RStudio Team) and filtered only ASVs that belonged to Bacteria and Archaea. Next, calculated the Effective Number of Species (ENS) [37] by calculating the exponential from the Simpson diversity index. We conducted SourceTracker analysis [38] to compare the proportions of bacterial taxa depletion and enrichment between neighbouring bulk soil and plant niches in healthy and unhealthy plantations. Core taxa for each niche were defined as those present in at least 95% of the samples and with relative relative abundance of at least 0.1%. The differential relative abundance analysis was conducted with Kruskal-Wallis test *p* value set to 0.05 and logarithmic LDA score to 2. Spearman correlations were determined in order to indicate which bulk soil chemical properties were significantly correlated with the bacterial biomarker taxa detected by LDA. We conducted Mantel correlation tests on matrices constructed from ASV table, geographic distance between farms, and chemical properties of the bulk soil with vegan library [39]. We quantified the assembly ecological processes that dominated microbial assembly in healthy and unhealthy strawberry farms, using RC_bray_ (Bray-Curtis dissimilarity based Raup-Crick metric) and βNTI (β Nearest Taxon) [40, 41]. In the case when the βNTI was > 2, community turnover would be dominated by heterogenous selection; when it was <-2, it would be dominated by homogenous selection. Next, the faction of pairwise comparisons between RC_bray_ >0.95 and |βNTI| <2 was considered to be dispersal limitation combined with drift. |βNTI| < 2 and |RCbray| < 0.95 was the influence of only drift, and |βNTI| < 2 and RCbray < -0.95 - homogenizing dispersal. We also utilized the PICRUSt2 (Phylogenetic Investigation of Communities by Reconstruction of Unobserved States) software [42] on a Linux system and the raw ASV table to predict Kyoto Encyclopedia of Genes and Genomes (KEGG) Ortholog (KO) relative abundances and determine KEGG pathway relative abundance. To identify the differentially abundant pathways between the healthy and unhealthy niches, we used Deseq2 algorithm [43].

## Results

### Bacterial community composition

A total of 165 soil (bulk soil and rhizosphere soil) and plant (root and shoot) samples were collected from 13 organic strawberry farms (cultivars: Aprica, Dipred, Honeoye) located on different soil types (Eutric Fluvisol, Eutric Cambisol, Mollic Gleysol, Haplic Luvisol, Arenic Fluvisol, Fluvic Gleysol) and analysed. After sequencing, we obtained 55,481 unique ASVs divided by 7 taxonomic ranks. The number of reads in each niche ranged from 141 in shoots to 127,381 in roots, with a mean of 42,462.11 and standard deviation of 29,669.27. The rarefaction curves reached a plateau in all of the samples, suggesting that bacterial diversity was adequately covered in our analysis (Supplementary Fig. 3). We used the mirlyn package to repeatedly rarefy the phyloseq objects for each niche independently [44–46] (Supplementary Fig. 3). Next, we compared the statistical differences between the relative abundance of the 15 most abundant bacterial phyla between healthy and unhealthy farms within the analyzed niches (Supplementary Fig. 4). Overall, the most abundant phyla were Proteobacteria, Actinobacteria, Bacteroidota, Acidobacteriota, and Verrucomicrobiota. In the bulk soil niche, only Acidobacteria and Verrucomicrobiota were statistically more abundant in unhealthy farms. In rhizosphere soil samples, Latescibacterota, Nitrospirota, and Verrucomicrobiota were more abundant in unhealthy samples. Finally, in root and shoot niches, there were no significant differences between the relative relative abundance of bacterial ASVs belonging to the 15 most abundant phyla between healthy and diseased farms.

### Microbiome of Strawberry plant niche exhibits the weakest correlation with bulk soil chemical properties

**Fig. 1:**
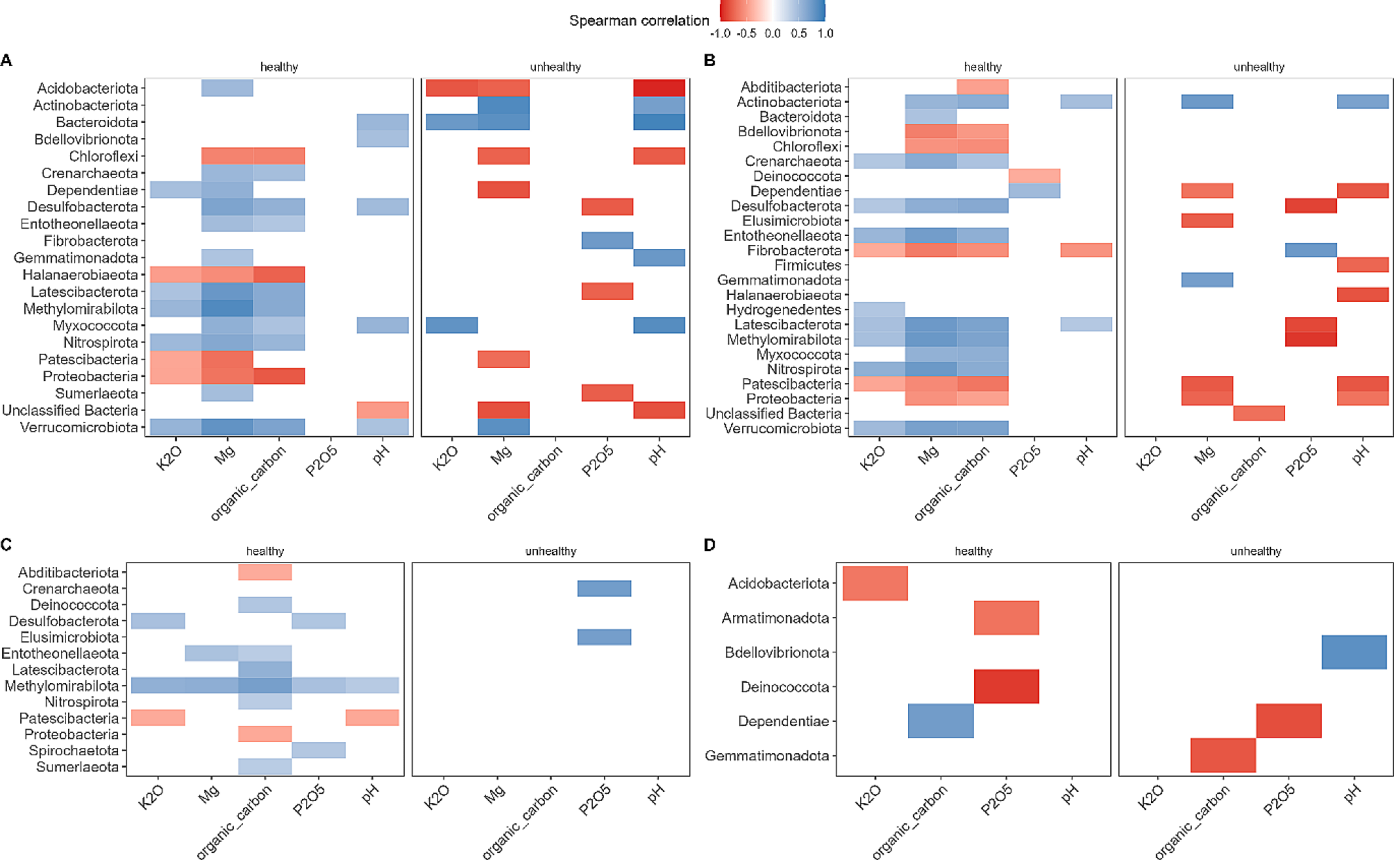
Significant Spearman correlations between mean bacterial phyla relative abundance in healthy and unhealthy plant and soil niches and chemical properties of the bulk soil samples. A. bulk soil, B rhizosphere soil, C roots, D shoots. The taxonomic nomenclature was based on the SILVA 138.1 database (2019)

In the subsequent stage of our research, our goal was to identify the relationships between the relative abundance of the major bacterial phyla and the chemical properties of the bulk soil in each niche independently. It was evaluated whether tested soil chemical properties were significantly different between healthy and unhealthy farms, and results suggested only weak evidence of higher concentration of Mg in unhealthy farms (Supplementary Fig. 5). We also observed that the bulk soil niche showed 21 bacterial phyla significantly correlated with the chemical properties of the bulk soil, and 41 for rhizosphere soil.Overall, healthy farms exhibited more phyla positively correlated with the chemical properties of the bulk soil in each niche, while unhealthy farms had more negative correlations between the relative abundance of bacterial phyla and the chemical contents in the bulk soil. Specifically, we identified 21 phyla in the bulk soil, 19 from healthy farms (14 positively and 5 negatively correlated) and 14 in unhealthy (6 positively and 8 negatively) (Fig. 1A).

In the rhizosphere soil niche, we found 24 significantly correlated phyla, 19 in healthy farms (12 positively and 7 negatively) and 12 in unhealthy (3 positively and 10 negatively) (Fig. 1B). In the root niche, we observed 13 significantly correlated phyla, 11 (8 positively and 3 negatively) in healthy and 2 (both positively) in unhealthy farms (Fig. 1C). Finally, the shoot niche was characterized by 6 significantly correlated phyla, 4 in healthy farms (1 positively and 3 negatively) and 3 in unhealthy (1 positively and 2 negatively) (Fig. 1D).

### The alpha and beta diversity indices exhibit comparable values in both healthy and diseased strawberry niches

**Fig. 2:**
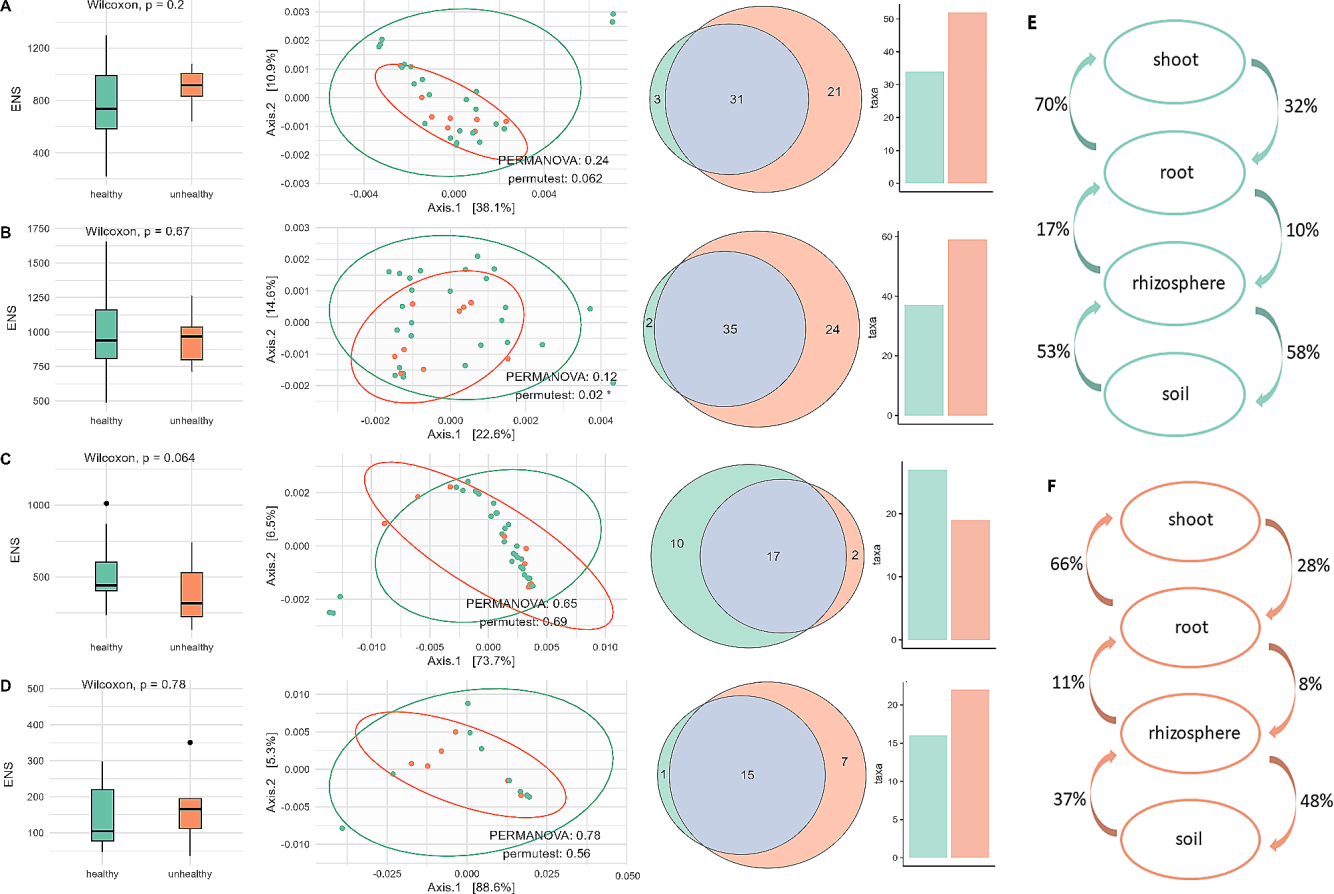
Alpha (Effective Number of Species - ENS) and beta diversity (PCoA plot of weighted UniFrac distances), venn diagrams, and bar plots presenting number of core taxa at ASV level of bacterial microorganisms found in healthy and unhealthy organic farms of strawberry analysed for A bulk soil, B rhizosphere soil, C root and D shoot niches. E and F represent the depletion and enrichment of bacterial microbiome between niches in healthy and unhealthy farms

We conducted statistical tests to compare the mean Effective Number of Species (ENS) [37] between healthy and unhealthy farms in each niche independently. The results indicated that there were no significant differences in the bacterial microbiomes between healthy and unhealthy farms in all niches (all p-values > 0.05). Notably, the shoot niche had the lowest ENS, followed by the root niche with a higher ENS, then the bulk soil niche, and finally the rhizosphere soil niche with the highest ENS (Fig. 2A).

We also performed β-diversity comparison between healthy and unhealthy farms in each niche using PERMANOVA (Fig. 2B). The β-diversity of the bacterial communities in the rhizosphere soil were the most complex within analysed niches, as the two PCoA axes explained 37.2% of the variation. The bulk niche diversity was explained in 49% by two axis, and roots and shoot in 80.2% and 93.3%, respectively. The results also showed that four niches did not have significant differences in the centroids (p-value > 0.05, Fig. 2), suggesting that the intra-variability between groups was higher than inter-variability. Additionally, betadisper analysis indicated that only the rhizosphere soil niche had the same dispersions between the health groups (p-value = 0.02).

### The root niche of unhealthy plants demonstrates the smallest number of core bacterial taxa and the highest specific beneficial bacteria in healthy roots

We further investigated the number of core taxa in healthy and unhealthy farms in each niche at the ASV level (Fig. 2). In the bulk soil niche, we found three core taxa for bulk soil collected from healthy farms, 21 for unhealthy, and 31 were common for both. In the healthy rhizosphere soil, two core taxa were identified, while unhealthy had 24, and 35 were common for both groups. In roots, samples from healthy farms had 10 core taxa, while unhealthy had only two, and 17 were common. Finally, in shoots, one core taxon was found in healthy farms, while unhealthy had seven, and 15 were common. The bar plots showed that the root niche was the richest in terms of number of core taxa in healthy farms (Fig. 2D). All names of core taxa are gathered and presented in the Supplementary Table 2.

### The rhizosphere soil microbiome of unhealthy farms demonstrates decline in migration from the bulk soil

We also revealed the enrichment and depletion of bacterial ASV between bulk soil and plant niches, comparing healthy and unhealthy farms (Fig. 2E). In both groups, the biggest percentage of bacterial ASVs migration was present between plant niches (root ◊ shoot: 70% in healthy and 66% in unhealthy) and then soil niches (rhizosphere soil ◊ bulk soil: 58% in healthy and 48% in unhealthy; bulk soil ◊ rhizosphere soil: 53% in healthy and 37% in unhealthy), in the same time showing weaker microbiome exchange between different environments (plant vs. bulk soil) through rhizosphere soil ◊ root (17% and 11% in healthy and unhealthy, respectively) and root ◊ rhizosphere soil (18% and 8% in healthy and unhealthy, respectively) directions. Interestingly, the exchange in microbiome between niches was more pronounced within healthy farms, and the biggest differences of bacterial migration between niches between healthy and diseased farms were visible within bulk soil niches (bulk soil ◊ rhizosphere soil and rhizosphere soil ◊ bulk soil, which were 16% and 10% bigger in healthy farms, respectively).

### Unhealthy farms exhibit an elevated relative abundance of bacterial health biomarkers

**Fig. 3:**
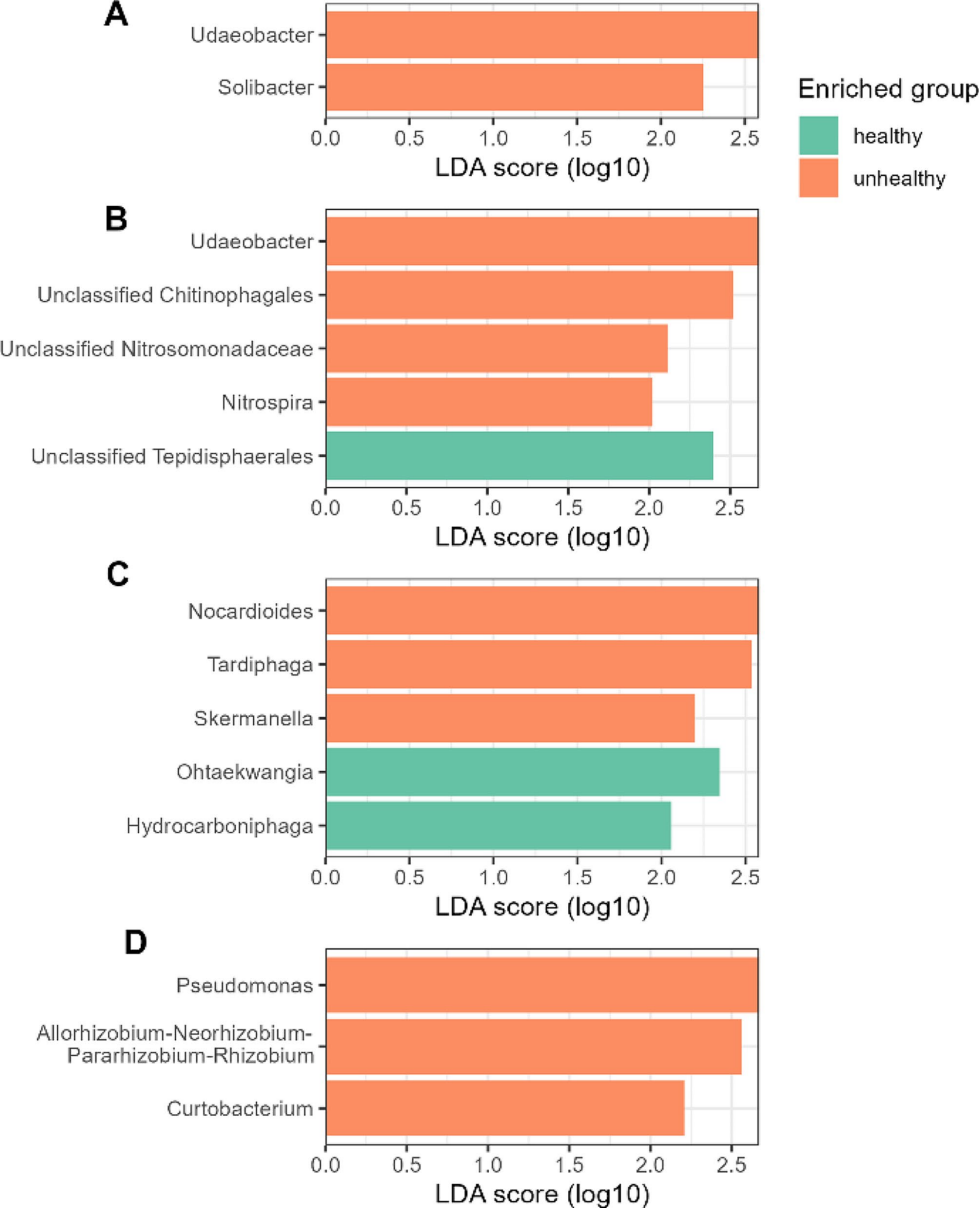
Bacterial biomarkers for healthy and unhealthy strawberry farms in 4 soil and plant niches (**A**: bulk soil, **B**: rhizosphere soil, **C**: roots, **D**: shoots), determined with LefSe. Different colors show different health groups.We also evaluated which soil chemical properties are significantly correlated with the relative abundance of taxa revealed by the biomarker identification (Supplementary Fig. 6). The analysis revealed that the relative abundance of *Udeobacter* spp. and unclassifies *Chitinophagales* was positively correlated with the Mg concentration in healthy and unhealthy farms. Also, we observed that the bacterial taxa from the rhizosphere were showed biggest number of correlations with the chemical properties of the soil

We used LDA effect size LefSe [47] for the health status biomarker identification. The analysis conducted for bulk soil and plant niches revealed, that bulk soil differed by 2 taxa (both unhealthy farms), followed by rhizosphere soil with 5 taxa (4 unhealthy and 1 healthy), then also 5 in roots (3 enriched in unhealthy farms and 2 in healthy), and only 3 characteristic for unhealthy shoots (LDA > 2, *p* < 0.05). Among these taxa, *Udaeobacter* sp. were characteristic for both, unhealthy bulk bulk soil and rhizosphere soil, and *Solibacter* sp. also being more abundant in unhealthy bulk soil (Fig. 3). The unhealthy rhizosphere soil, next to enriched *Udaeobacter*, also revealed more abundant unclassified Chitinophagales, unclassified Nitrosomonadaceae, *Nitrospira* sp., and in healthy farms - unclassified Tepidisphaerales. Root niche also revealed 5 enriched taxa - in unhealthy farms: *Nocardioodes* sp., *Tardiphaga* sp. and *Skemanella* sp., and in healthy: *Ohtaekwangia* sp. and *Hydrocarboniphaga* sp. Finally, shoot samples were characterized with enrichment of 3 taxa in unhealthy farms: *Pseudomonas* sp., *Allorhizobium-Neorhizobium-Pararhizobium-Rhizobium* sp. and *Cutobacterium* (Fig. 3).

### Shoot niche demonstrated no significant positive correlations between bacterial alpha-diversity and bulk soil chemical properties

**Fig. 4:**
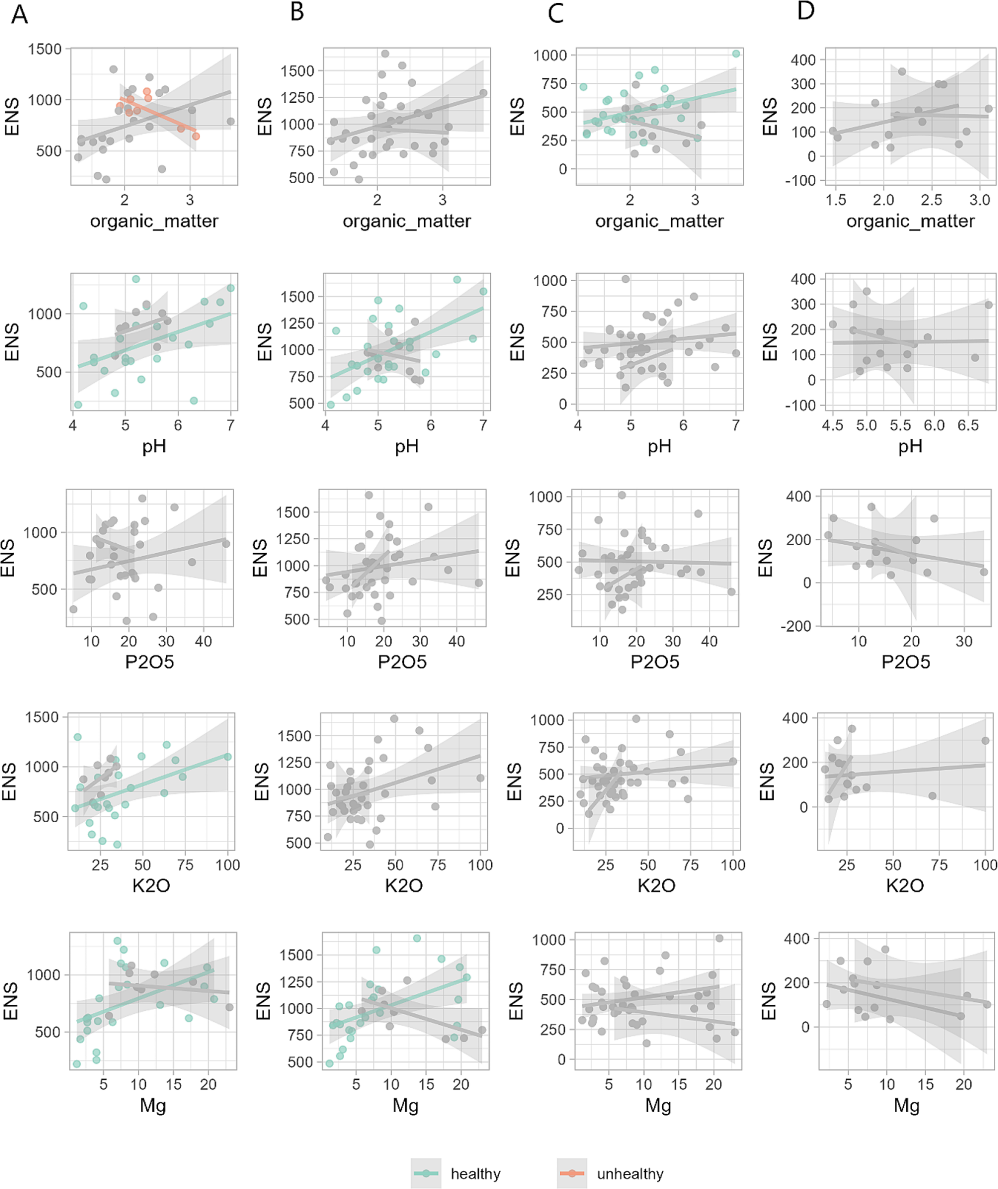
The relationship between chemical properties of the soil in healthy farms and alpha diversity (in Effective Number of Species - ENS) of bacterial communities in each niche. The lines and shaded areas represent the relationship with 95% confidence intervals for the interaction revealed with linear model. **A** - bulk soil, **B** - rhizosphere soil, **C** - roots, **D** - shoots. Significant relationships (*p* > 0.05) are showed in green (for healthy farms) and red (for unhealthy), whereas not significant (*p* ≤ 0.05) in grey. Adjusted R^2^ values for significant correlations are: (A) organic matter: 0.1134, pH: 0.1526, K_2_O: 0.1557, Mg: 0.1834; (**B**) pH: 0.2775, Mg: 0.2274; (**C**) organic matter: 0.1095

In the next stage of our research, we aimed to investigate the relationship between bulk soil chemical properties and the alpha diversity of bacterial microbiomes in each niche of healthy and unhealthy farms with a linear regression model (Fig. 4).

The results indicated that P_2_O_5_ contents did not show any significant relationship with bacterial ENS in any niche. However, pH and Mg were found to be positively correlated with ENS in both healthy bulk soil niches, and K_2_O contents were positively correlated with ENS in the healthy bulk soil. Furthermore, organic matter contents showed a negative correlation with ENS in the unhealthy bulk soil niche, and a positive correlation with ENS in the root niche of healthy farm.

### Deterministic processes drive microbial assembly in unhealthy bulk soil, rhizosphere soil and roots, but not in shoots

We used Mantel test to evaluate the association between the microbiome of strawberry plantations, theirs geographical distance and chemical properties of the bulk soil. The results outline, that the geographic distance have a strong relationship with the species Bray-Curtis dissimilarity matrix of bacteria, in bulk soil and rhizosphere soil in both, healthy and unhealthy farms (Table 1). On the other hand, cumulative chemical bulk soil properties showed correlation only with bulk soil microbial communities in healthy farms (Mantel statistic 0.28, *p* = 0.007), suggesting distance decay pattern in microbial communities, and comparatively big dispersal limitation, at least in rhizosphere soil samples.

**Fig. 5:**
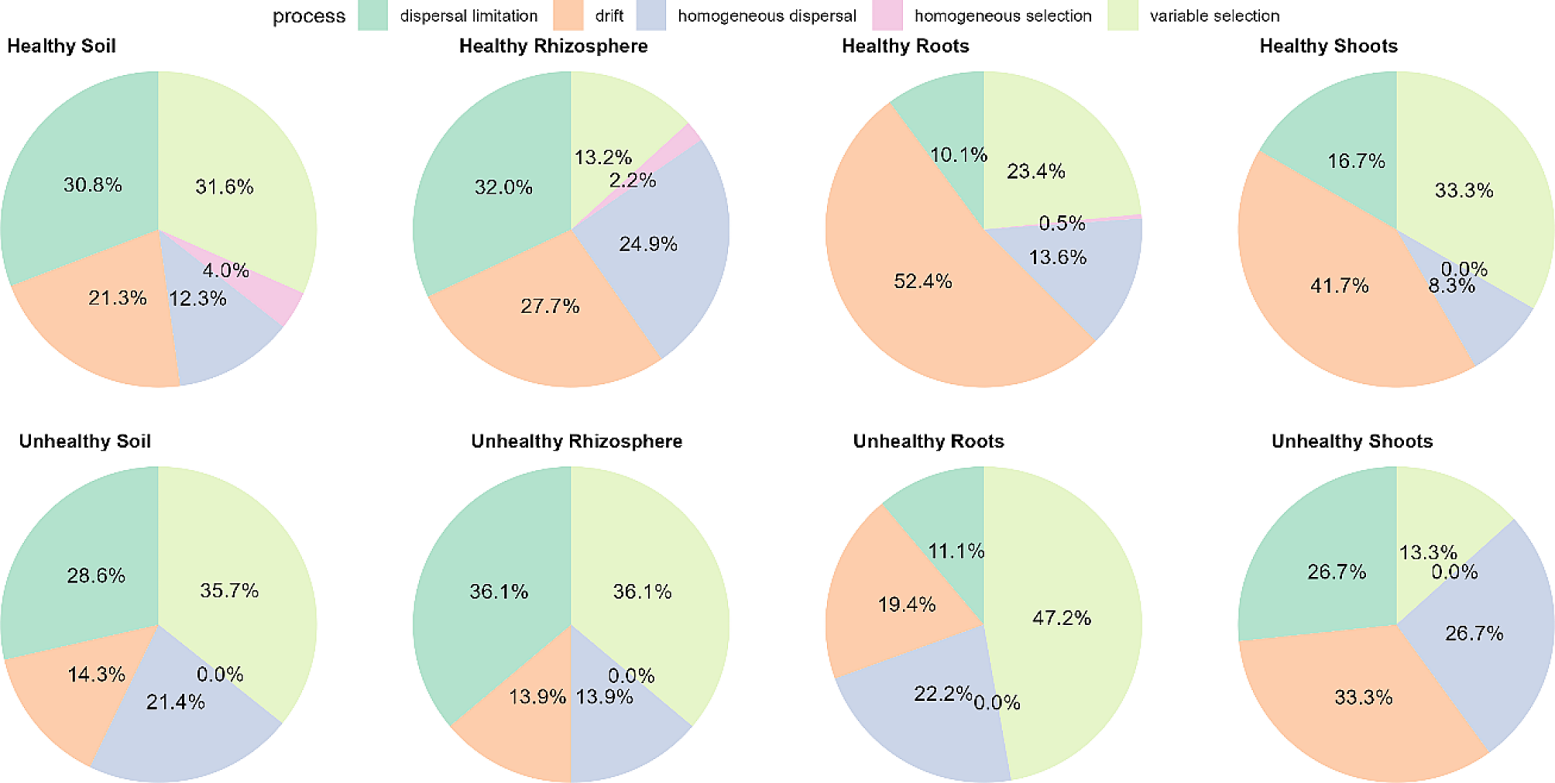
Pie chart representing percentage of processes on community assembly calculated with betaNTI and RCbray analysis of null model

We then calculated the proportions of community assembly processes for healthy and unhealthy farms [30, 31]. The analysis revealed increased share of variable selection (high microbial turnover caused by shifts in environmental factors) in bulk soil, rhizosphere soil and roots in unhealthy farms (Fig. 5).

**Table 1:** Mantel test results determining the correlation between ASV relative abundance dissimilarity matrix and geographic distance or chemical properties of the bulk soil matrix. Significant results are showed in bold

	Geographical distance				Chemical properties of bulk soil			
	Healthy farms		Unhealthy farms		Healthy farms		Unhealthy farms	
	Mantel statistic	P	Mantel statistic	P	Mantel statistic	P	Mantel statistic	P
Bulk soil	**0.5**	**1e-04**	**0.77**	**0.002**	**0.28**	**0.007**	0.48	0.02
Rhizosphere soil	**0.29**	**1e-04**	**0.9**	**5e-04**	0.48	0.02	0.30	0.07
Roots	0.07	0.07	0.19	0.12	0.3	0.07	0.03	0.44
Shoots	0.11	0.16	0.16	0.32	0.03	0.44	0.36	0.14

Interestingly, homogeneous selection (low microbial compositional turnover caused by consistent environmental factors) was the least important ecological process, not present in unhealthy farms. Stochastic processes - homogenous dispersal and drift were decreased in unhealthy rhizosphere soil and roots, but similar to healthy bulk soil and increased in unhealthy shoots. Finally, dispersal limitation was similar in both groups - healthy and unhealthy farms, and was the most important in the rhizosphere soil, as we concluded with Mantel test.

What is more, we observed plant niches differences in functional pathways between healthy and unhealthy farms. Overall, 7 most abundant subclasses that were detected in 80 − 35% of samples were: carbohydrate metabolism, amino acid metabolism, metabolism of cofactors and vitamins, metabolism of terpenoids and polyketides, xenobiotics biodegradation and metabolism, lipid metabolism and energy metabolism from metabolism KEGG class (Supplementary Fig. 7A). The predicted pathways also consisted classes, such as cellular processes, environmental information processing, organismal systems and genetic information processing. The analysis of predicted functional pathways relative abundances between niches in healthy and unhealthy farms indicated that bulk soil, root and shoot niches showed no significant differences between two health groups (Supplementary Fig. 7B, 7D, 7E), while rhizosphere soil exhibited differences. Namely, pathways related to the naphthalene degradation and indole alkaloid biosynthesis were less abundant in healthy farms, compared to unhealthy (Supplementary Fig. 7C).

## Discussion

In the present study, we investigated the differences in microbiome characteristics within four plant and soil niches (bulk soil, rhizosphere soil, roots, and shoots) and their relationship with the chemical properties of the bulk soil in healthy and unhealthy organic strawberry farms. We conducted sequencing of the 16 S V3-V4 region to taxonomically identify the bacteria in each niche. Subsequently, we performed bioinformatic and statistical analyses to reveal significant differences in microbiome structure between healthy and unhealthy strawberry farms.

Consistent with previous studies [48–50], the most abundant bacterial phyla in the bulk soil belonged to Proteobacteria, Actinobacteria, Acidobacteria, and Verrucomicrobiota. In our study, we also identified Bacteroidota as one of the most abundant bacterial groups. Interestingly, these most abundant phyla were significantly correlated with the chemical properties of the bulk soil. For instance, the relative abundance of Proteobacteria was strongly negatively correlated with K_2_O, Mg, and total organic carbon contents in the bulk soil of healthy farms. In the rhizosphere soil niche, this taxon was also strongly negatively correlated with Mg and total organic carbon contents, whereas in roots, it was only correlated with organic carbon contents. On the other hand, Bacteroidota showed a strong positive correlation with the pH of unhealthy bulk soil niche, but also with Mg and pH in the bulk soil of healthy farms. Acidobacteriota, as the next most abundant taxon, exhibited positive correlation with Mg contents in healthy bulk soil and strong negative correlations with K_2_O, Mg, and pH in unhealthy plantations. In the healthy rhizosphere soil niche, Acidobacteriota showed a negative correlation with organic carbon, and in the shoot niche indicated a negative correlation with K_2_O contents. Amongst these most abundant phyla, Verrumicrobiota showed the most correlations with the chemical properties of the bulk soil - it revealed positive correlations with K_2_O, Mg, organic carbon, and pH in healthy bulk soil, and only with Mg in unhealthy bulk soil.

It had been reported, that α-diversity of microbial communities play an important role in the plant disease resilience, especially in the rhizosphere soil [51], as roots release chemicals that recruit beneficial microorganisms [16, 52]. In contrast to these results, our findings did not report any significant differences in the α-diversity of the bacterial microbiome neither in rhizosphere soil, nor other niches (bulk soil, roots and shoots). However, ecological process analysis revealed increased variable selection in all unhealthy plant parts - rhizosphere soil and roots, suggesting selective pressure of strawberry plant in disease conditions - “cry for help” [53]. Interestingly, in unhealthy shoots, we observed increased share of stochastic processes, showing that the unhealthy microbial assembly in less predictable than in healthy farms, showing Anna Karenina Principle [21]. This could be caused by the reduction of strawberry immune response to the pathogen colonization, simultaneously allowing colonization of different, random microorganisms [54, 55]. Overall, in unhealthy farms, a share or deterministic processes of bacterial assembly in rhizosphere soil and root was increased (15,4% vs. 36,1%; 23,9% vs. 47,2%), suggesting anti-AKP process [21]. This could be caused by either, the recruitment of beneficial microorganisms by plant, or strong environmental filtering occurring in these niches under stress conditions. We also noted decreasing α-diversity from rhizosphere soil, through bulk soil, roots and shoots, and decreasing number of bacterial phylas correlated with chemical properties of the soil from bulk soil to shoots, suggesting a selective influence of the strawberry host on bacterial communities found in the bulk soil [56].

Beta diversity analysis revealed no separate clusterings in the bulk soil and plant niches when it comes to the health status of the strawberry farms, similarly to the study conducted on tomatoes [57]. These results prompted us to conduct a more in-depth analysis to identify distinct differences in the microbiome associated with healthy and diseased strawberry plantations. As a result, we identified the core taxa for each of the bulk soil and plant niches. As expected, the rhizosphere soil niche showed the highest number of core taxa (61), followed by bulk soil (55), roots (29), and shoots (23). The most typical and unique core taxa for healthy niches included Pseudarthrobacter sp. and Streptomyces sp. for bulk soil, Unclassified Vicinamibacterales, Unclassified Thermomicrobiales and Unclassified Planctomycetales for rhizosphere soil, as well as Unclassified Acidimicrobiia and Unclassified Micropepsaceae for both bulk and rhizosphere soil. In healthy strawberry shoots only Microbacterium sp. was specific for healthy plants, while in roots appered the following specific core taxa Actinoplanes sp., Unclassified Micromonosporaceae, Streptomyces sp., Unclassified Microscillaceae, Bacteria_ Bacteroidota_ Bacteroidia_ Flavobacteriales_ Flavobacteriaceae_ Flavobacterium_ Flavobacterium sp., Mucilaginibacter sp., Unclassified Thermomicrobiales, Methylobacterium sp., Mesorhizobium sp., Rhizobacter sp. and Unclassified Saccharimonadales. The analysis also revealed that the root niche of healthy strawberry farms was characterized by the largest group of core taxa, indicating that the root is an important source of crucial microorganisms [26, 58].

We then identified potential bacterial biomarkers of strawberry health status. *Udaeobacter* sp., ubiquitous bulk soil bacteria [59], was the most significantly enriched taxon in the analyzed unhealthy bulk soil and rhizosphere soil niches. It was also reported to be sensitive to microplastic exposure [60], and benefited from antibiotic pollution of the bulk soil [61]. Additionally, we found that *Solibacter* sp., enriched in unhealthy strawberry bulk soil, was reported as an important taxon in forming the wheat rhizosphere soil, containing crucial functional genes related to carbon (C), nitrogen (N), and phosphorus (P) cycling [62]. This taxon was also enriched in *Rhizoctonia solani*-infected potato rhizosphere soil [63]. In the rhizosphere soil niche, alongside *Udaeobacter* sp., we identified that unclassified Chitinophagales were enriched in unhealthy niches. Bacteria belonging to this taxon were described as capable of metabolizing lignin or chitin [64]. The analysis also revealed important biomarkers of unhealthy rhizosphere soil - unclassified Nitrosomonadaceae and *Nitrospira* spp., which belong to nitrifying bacteria [65] and Chitinophagales, which were reported to be abundant in microplastic-contaminated bulk soil [66] and positively correlated with the relative abundance of functional genes - *amoA* and *amoB* [62]. These results suggest that the presence of these taxa is an important indicator of the plant host’s fitness. Continuing, Unclassified Tepidisphaerales, thermophilic bacteria [67], were an important indicator in healthy rhizosphere soil samples. Next, important unhealthy root biomarkers were identified including the following genera - *Nocardioides*,* Tardiphaga* and *Skemanella* belonging to actinobacteria, as well as Ohtaekwangia and Hydrocarboniphaga, in healthy samples. Proceeding to the shoot niche, pathogen-suppressing *Pseudomonas* sp. was an important biomarker in the unhealthy shoot niche, significantly negatively correlated with the disease incidence of tomatoes [68]. Another unhealthy farm biomarker, N_2_-fixing bacteria, *Allorhizobium-Neorhizobium-Pararhizobium-Rhizobium* sp., were identified as potential keystone taxa in microplastic-contaminated bulk soil in forests [69]. On the other hand, *Curtobacterium* sp. were reduced in atmospheric cold plasma-treated blueberries [70], but they were also reported to exhibit plant-promoting activity [71].

The analysis also revealed that organic matter content, pH, and Mg were the most significant factors correlated with alpha diversity of bacterial communities in both healthy and unhealthy strawberry farms, consistent with findings from other studies on different plants [72–74]. Additionally, the microbiomes in roots and shoot niches showed less dependence on the chemical properties of the bulk soil, except for the organic matter content in the roots, where it was directly proportional to the Effective Number of Species in this niche. Bulk soil organic carbon (organic matter) serves as the primary nutrient and energy source for the microbiomes, constituting essential environmental factors that influence microbial composition and host plant health [68]. In summary, our analysis revealed that bulk soil chemical properties were significantly related to the alpha diversity of bacterial microbiomes in several niches. Specifically, we found that organic matter, pH, K_2_O, and Mg were significantly related to alpha diversity in the bulk soil niche, while pH and Mg were related to alpha diversity in the rhizosphere soil niche. Additionally, organic matter was related to alpha diversity in the root niche of healthy farms. Interestingly, we found no significant relationships between bulk soil chemical properties and alpha diversity in the shoot niche, suggesting disconnection of the microbiome of the shoot from the bulk soil chemical properties.Regarding the differential abundance of predicted functional pathways of microbiota present in the analyzed niches, there were no significant differences between healthy and unhealthy bulk soil, roots and shoots. However, rhizosphere soil showed differences in the composition of functional pathways. In unhealthy roots, the naphthalene degradation pathway was decreased compared to healthy farms. Naphthalenes exhibit genotoxic and carcinogenic effects on living organisms [68]. This predicted activity suggests that unhealthy farms could be contaminated with these substances, and unhealthy plants recruited microbiota responsible for their degradation. Additionally, in unhealthy roots, indole alkaloid biosynthesis was enriched - the alkaloids isolated from Pseudomonas aeruginosa have been known to exhibit antibacterial activity [75]. It is possible that some of the bacteria from unhealthy roots produced these substances to prevent the recruitment of the bulk soil microbiome by the host plant.

## Conclusion

The research showed, that in healthy bulk soil Acidobacteria and Verrucomicrobiota and in rhizosphere soil - Latescibacterota, Nitrospirota, and Verrucomicrobiota, were more abundant than in unhealthy, whereas plant niches (root and shoot) showed no statistically significant differences in bacterial relative abundance. Importantly, we also revealed the ecological processes that shape microbial assembly in healthy and unhealthy farms - increased deterministic processes in unhealthy rhizosphere soil and roots, suggesting ‘cry for help’ mechanism in strawberry plant. We observed that healthy farms demonstrated highest relative abundance of bacterial phyla that exhibited positive correlations with the chemical properties of the bulk soil. In contrast, unhealthy farms displayed an increased prevalence of negative correlations between the relative abundance of bacterial phyla and the chemical composition of the bulk soil. Meanwhile, there were no significant differences between healthy and unhealthy farms in terms of alpha diversity, nonetheless, we identified a decreasing alpha diversity trend from the rhizosphere soil through bulk soil, roots, and shoots. This suggests that the strawberry host exerts selective influences on bacterial communities found in the bulk soil, which may contribute to plant health. The unhealthy rhizosphere soil revealed the highest number of core taxa amongst all evaluated niches, at the same time showing the biggest differentiation of bacterial ASVs migration between healthy and unhealthy farms in rhizosphere soil → bulk soil and bulk soil → rhizosphere soil directions. This highlighted the rhizosphere soil’s critical role as a reservoir for beneficial microorganisms, essential for plant health. The study also revealed that only shoot niche showed no significant correlations between α-diversity of microbiomes and evaluated bulk soil chemical properties. Additionally, we identified potential bacterial biomarkers associated with strawberry health status, including eubiotic biomarkers: Unclassified Tepidisphaerales, Ohtaekwangia, Hydrocarboniphaga and dysbiotic biomarkers: Udaeobacter, Solibacter, Unclassified Chitinophagales, Unclassified Nitrosomonadaceae, Nitrospira, Nocardioides, Tardiphaga, Skermanella, Pseudomonas, Allorhizobium-Neorhizobium-Pararhizobium-Rhizobium, Curtobacterium, potentially important in the strawberry soterobiont concept.

Our study aimed to improve the understanding the context of organic bulk soil health status and plant-microbiome interactions in strawberry farms. By understanding the roles of various bacterial taxa and environmental factors, we can further develop strategies to improve plant health and disease resilience in sustainable agricultural practices. Contributing to this growing body of knowledge by shedding a light on the bacterial community composition in bulk soil and plant niches of healthy and unhealthy strawberry farms can improve the understanding of sustainable agriculture. The data obtained may be a guideline for the development of important solutions for 4.0. Agriculture, based on artificial intelligence and microbiome research for the prediction and monitoring of bulk soil and plant condition in strawberry cultivation.

### Electronic supplementary material

Below is the link to the electronic supplementary material.

Supplementary Material 1

## Data Availability

All data generated and analyzed during this study are included in this published article and its supplementary information files. The raw DNA sequencing data generated in the study are available at the Sequence Read Archive (SRA) database in National Center for Biotechnology Information (NCBI) with the accession number PRJNA1073294 (https://www.ncbi.nlm.nih.gov/bioproject/PRJNA1073294).

## Abbreviations

ISR: Induced systemic resistance
SAR: Systemic acquired resistance
QIIME2: Quantitative Insights Into Microbial Ecology
ASVs: Amplicon Sequence Variants
DADA2: Divisive Amplicon Denoising Algorithm v. 2
ENS: Effective Number of Species
LDA: Linear Discriminant Analysis
RCbray: Bray-Curtis dissimilarity based Raup-Crick metric
βNTI: βNearest Taxon
PICRUSt2: Phylogenetic Investigation of Communities by Reconstruction of Unobserved States
KEGG: Kyoto Encyclopedia of Genes and Genomes
KO: Ortholog
